# Durvalumab in frail and elderly patients with stage four non-small cell lung cancer: Study protocol of the randomized phase II DURATION trial

**DOI:** 10.1186/s13063-020-04280-8

**Published:** 2020-04-22

**Authors:** Jonas Kuon, Adriane Hommertgen, Johannes Krisam, Felix Lasitschka, Albrecht Stenzinger, Miriam Blasi, Farastuk Bozorgmehr, Martin Maenz, Meinhard Kieser, Marc Schneider, Michael Thomas

**Affiliations:** 1 Heidelberg University Hospital, Department of Thoracic Oncology, Röntgenstraße 1, 69126 Heidelberg, Germany; 2 German Cancer Research Center, Abteilung für Molekulare Radioonkologie, Im Neuenheimer Feld 280, 69120 Heidelberg, Germany; 3 Heidelberg University Hospital, Institute of Medical Biometry and Informatics, Im Neuenheimer Feld 130.3, 69120 Heidelberg, Germany; 4 Heidelberg University Hospital, Institute of Pathology, Im Neuenheimer Feld 430, 69120 Heidelberg, Germany; 5 Translational Lung Research Center Heidelberg TLRC-H, Member of the German Center for Lung Research DZL, Im Neuenheimer Feld 156, 69120 Heidelberg, Germany; 6 AIO-Studien-gGmbH, Berlin, Germany; 7 Heidelberg University Hospital, Translational Research Unit, Röntgenstraße 1, 69126 Heidelberg, Germany

**Keywords:** Lung cancer, Durvalumab, PD-L1, Elderly, Frail, CARG

## Abstract

**Background:**

Elderly patients represent a major fraction of non-small cell lung cancer (NSCLC) patients in routine clinical practice, but they are still underrepresented in clinical trials. In particular, data regarding efficacy and safety in frail or elderly patients with respect to immunotherapy are lacking. Importantly, immunosenescence in elderly patients might interfere with activities of immune-modulating drugs such as PD-1/PD-L1 inhibitors. Thus, there is an urgent need to assess safety and efficacy of such inhibitors in this group.

**Methods/design:**

This prospective, open label, treatment stratified, randomized phase II study will enroll 200 patients with stage IV NSCLC amenable at least to single-agent chemotherapy (CT). Eligible patients must be aged 70 years or older and/or “frail” (Charlson Comorbidity Index > 1) or have a restricted performance status (Eastern Cooperative Oncology Group, ECOG > 1).

Patients are stratified according to modified Cancer and Age Research Group (CARG) score: “fit” patients are allocated to combination CT (carboplatin/*nab*-paclitaxel) and “less fit” patients receive single-agent CT (gemcitabine or vinorelbine). After allocation to strata, patients are randomized 1:1 to receive either four cycles of CT or two cycles of CT followed by two cycles of durvalumab and subsequent maintenance treatment with durvalumab every 4 weeks.

The primary endpoint is the rate of treatment-related grade III/IV adverse events (Common Terminology Criteria for Adverse Events (CTCAE) V4.03). As secondary endpoints, progression-free survival (PFS) according to Response Evaluation Criteria in Solid Tumours (RECIST) version 1.1, response rate (RR), overall survival (OS), descriptive subgroup analyses according to PD-L1 expression, and quality of life are addressed. Geriatric screening assessments and functional tests will be performed to complete the phenotyping of a potential “frail” and “elderly” patient cohort. The trial is accompanied by a biomaterial repository to explore potential biomarkers.

**Discussion:**

The DURATION trial will prospectively investigate the safety and tolerability of anti-PD-L1 treatment with durvalumab after chemotherapy in elderly and frail patients and thereby provide new insights into the effect of PD-L1 blockade and the impact of immunosenescence in this cohort of patients.

**Trial registration:**

ClinicalTrials.gov, NCT03345810; initially registered on 17 November 2017.

Eudra-CT, 2016–003963-20; initially registered on 3 January 2017.

## Background

Lung cancer is one of the most commonly diagnosed cancer types and the leading cause of cancer death worldwide [1]. It is predominantly a disease of the elderly, with about 50% of patients diagnosed aged 70 years or older [2]. Age is an independent prognostic factor affecting survival in this group of patients [3].

Metastatic non-small cell lung cancer (NSCLC) carries a dismal prognosis with a median survival of less than 12 months [4]. Systemic treatment, such as mono-chemotherapy (e.g., vinorelbine or gemcitabine) [5, 6] or a dose-adapted combination of carboplatin (area under the curve (AUC) 6)/paclitaxel (90 mg/m2; d1 + 8 + 15) [7], is considered to improve outcomes in patients who are aged ≥ 70 years, are frail, or have a reduced performance score (i.e., Eastern Cooperative Oncology Group (ECOG) ≥ 2).

The use of geriatric assessment and screening tools is recommended in elderly patients to better adapt intensity of treatment to patient condition and comorbidities [8]. As a complex geriatric assessment (CGA) is time- and resource-consuming and potentially not required for all patients, shorter pre-therapy risk assessments have been developed, i.e., the CRASH (Chemotherapy Risk Assessment Scale for High-Age Patients) [9] and the CARG (Cancer and Age Research Group) [10] scores.

While the CRASH score contains variables for clinical and geriatric assessment, the CARG score comprises geriatric assessment questions and clinical questions concerning items retrieved from everyday practice. Both scores are considered as useful toxicity prediction tools, appropriate for implementation in routine clinical practice, with a potential impact on the optimization of therapy selection for elderly patients with cancer [11].

With the advent of immune-oncology, new options have become available. Currently, checkpoint inhibition therapies targeting PD-1 and PD-L1 are established as first-line treatment in metastatic lung cancer. This is based on several impressive efficacy results of anti-PD-1/PD-L1 antibodies in clinical trials in NSCLC that have led to the approval of nivolumab, pembrolizumab, and atezolizumab [12–15] first in advanced therapy lines and later in the first-line setting (mono-immunotherapy for PD-L1 ≥ 50% or regardless of PD-L1 tumor expression in combination with a combination chemotherapy) [16, 17]. However, clinical evidence about the tolerability and safety of checkpoint inhibition as a treatment option in frail and elderly patients is still lacking.

In addition, it remains an unresolved question whether efficacy of checkpoint inhibitors in older patients is affected by a still poorly characterized phenomenon referred to as immunosenescence, i.e., the global and progressive remodeling of immune functions with aging [14, 15, 18, 19]. Age-related alterations such as impaired T-cell activation, reduced T-cell receptor diversity, altered antigen-uptake and -presenting functions, or the increased generation of immune suppressive cells may impair the antitumor response and thus could be one of the reasons for a higher incidence and prevalence of most cancers in older people.

Furthermore, a paradoxical stimulation of tumor growth upon initiation of treatment with checkpoint inhibitors, so-called hyperprogressive disease, has been reported in a recent retrospective analysis in up to 19% of patients older than 65 years. This could potentially be caused by an altered immune function due to immunosenescence [20].

In conclusion, with the increasing use of immunotherapy in everyday clinical practice there is a growing interest in immunosenescence and how it may correlate with outcomes of immunotherapy in elderly patients [18, 21]. However, data derived from randomized trials investigating safety and tolerability of checkpoint inhibitors in this specific population of lung cancer patients are lacking.

## Methods/design

### Study design

**Fig. 1:**
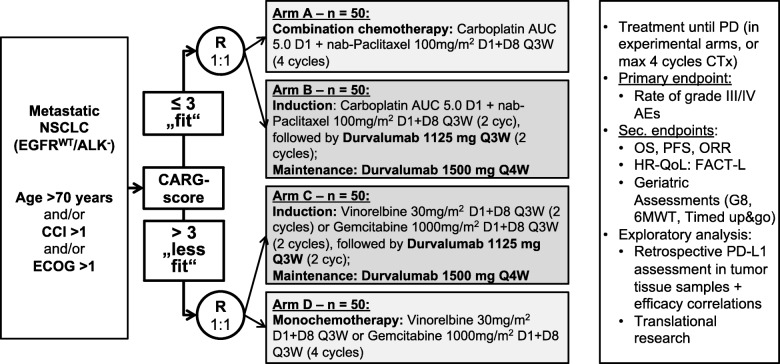
DURATION patient allocation and treatment strategy

The DURATION study is an open label, treatment-stratified, and randomized phase II study (Fig. 1) enrolling patients with histologically confirmed NSCLC (adenocarcinoma and squamous) stage four (metastatic) prior to any systemic treatment. All procedures and time frames displayed in Fig. 1 and the schedule of assessment (Table 3) were developed according to the Standard Protocol Items: Recommendations for Interventional Trials (SPIRIT). Additional file 1 contains the complete SPIRIT checklist.

### Study setting

The DURATION trial is a multicenter trial, recruiting patients from approximately 30 sites across Germany. A full list of sites can be obtained at ClinicalTrials.gov (NCT03345810).

### Study objectives

#### Primary objective

The primary objective is to investigate the safety and tolerability of sequential therapy consisting of standard of care single agent or doublet chemotherapy followed by durvalumab in comparison to standard of care single-agent or doublet chemotherapy in frail and/or elderly patients.

#### Secondary objectives

Secondary objectives are to collect information on efficacy, safety, and quality of life parameters and to investigate the utility of geriatric assessments for treatment guidance. Geriatric assessment and quality of life parameters will be collected before and at distinct time points during the treatment course.

#### Exploratory objectives

Exploratory objectives are to identify potential predictive biomarkers for efficacy variables. To this end, tissue collection and blood sampling will be performed before and during the course of disease/treatment. The blood and tissue samples will be subjected to molecular analyses to search for markers of immune response in this population.

Analysis of biomarker data will include correlations with clinical phenotype and tumor PD-L1 expression.

### Characteristics of patients

Two-hundred frail and/or elderly patients with metastatic NSCLC with no targetable molecular alterations (*EGFR*^wt^, *EML4ALK*^transl-^) before first-line treatment will be included.

Key inclusion criteria are age ≥ 70 years and/or Charlson Comorbidity Index (CCI) > 1 and/or ECOG > 1, previously untreated NSCLC with no targetable molecular alterations (*EGFR*^wt^, *EML4ALK*^transl-^), and the availability of a formalin-fixed, paraffin-embedded (FFPE) tumor tissue block (fresh or archival less than 3 years old or recent) or a minimum of ten unstained slides of tumor sample for biomarker (PD-L1) evaluation. Key exclusion criteria include mixed small cell lung cancer with NSCLC and large-cell lung carcinoma histology and history of another primary active malignancy or active autoimmune disease.

For a full list of inclusion and exclusion criteria see Table 1.

**Table 1:** Complete list of inclusion/exclusion criteria

**Inclusion criteria**	
• Written informed consent and any locally required authorization (EU Data Privacy Directive in the EU) obtained from the subject prior to performing any protocol-related procedures, including screening evaluations • Aged ≥ 70 years at time of study entry and/or Charlson-Comorbidity-Index (CCI) > 1 and/or performance status PS > 1 • Histologically confirmed diagnosis of metastatic NSCLC and no targetable molecular alterations (EGFR WT; ALK transl-) • Patients with measurable disease according to Response Evaluation Criteria in Solid Tumors (RECIST 1.1) • A formalin fixed, paraffin-embedded (FFPE) tumor tissue block or a minimum of ten unstained slides of tumor sample • No prior chemotherapy or any other systemic therapy for metastatic NSCLC. Patients who received prior platinum-containing adjuvant, neoadjuvant, or definitive chemoradiation for locally advanced disease are eligible, provided that progression has occurred > 6 months from last therapy • Prior radiotherapy and surgery are allowed if completed 4 weeks prior to start of treatment and patient recovered from toxic effects or associated adverse events • Adequate blood count, liver-enzymes, and renal function ◦ Hemoglobin ≥ 9.0 g/dl ◦ Absolute neutrophil count (ANC) ≥ 1.5 × 10^9^/L (> 100 per mm^3^) ◦ Platelet count ≥ 100 × 10^9^/L (> 100,00 per mm^3^) ◦ Serum bilirubin ≤ 1.5 × ULN. This will not apply to subjects with confirmed Gilbert’s syndrome (persistent or recurrent hyperbilirubinemia that is predominantly unconjugated in the absence of hemolysis or hepatic pathology), who will be allowed only in consultation with their physician ◦ AST (SGOT)/ALT (SGPT) ≤ 2.5 × institutional upper limit of normal unless liver metastases are present, in which case it must be ≤ 5 × ULN ◦ Serum creatinine CL > 40 mL/min by the Cockcroft-Gault formula (Cockcroft and Gault 1976) or by 24-h urine collection for determination of creatinine clearance ◦ Subject is willing and able to comply with the protocol for the duration of the study including undergoing treatment and scheduled visits, examinations including follow-up, and appropriate contraception	
**Exclusion criteria**	
• Mixed small-cell lung cancer with NSCLC and large-cell lung cancer histology • Mean QT interval corrected for heart rate (QTc) ≥ 470 ms calculated from three electrocardiograms (ECGs) using Fredericia’s correction • History of another primary malignancy except local prostate cancer without need for systemic treatment (e.g., active surveillance, operation without need for adjuvant treatment) and malignancies treated with curative intent and with no known active disease > 2 years before the first dose of study drug and of low potential risk for recurrence—adequately treated non-melanoma skin cancer or lentigo maligna without evidence of disease—adequately treated carcinoma in situ without evidence of disease (e.g., cervical cancer in situ) • Pre-existing peripheral neuropathy of grade ≥ 2 • Brain metastasis or spinal cord compression unless asymptomatic or treated and stable off steroids and anticonvulsants for at least 1 month prior to study treatment. • Active or prior documented autoimmune disease within the past 2 years. Note: Subjects with vitiligo, Grave’s disease, or psoriasis not requiring systemic treatment (within the past 2 years) are not excluded • Active or prior documented inflammatory bowel disease (e.g., Crohn’s disease, ulcerative colitis) • History of primary immunodeficiency • History of allogeneic organ transplant • History of hypersensitivity to durvalumab or any excipient • History of hypersensitivity to any of the comparator agents • Medication that is known to interfere with any of the agents applied in the trial • Uncontrolled intercurrent illness including, but not limited to, ongoing or active infection, symptomatic congestive heart failure, uncontrolled hypertension, unstable angina pectoris, cardiac arrhythmia, active peptic ulcer disease or gastritis, active bleeding diatheses including any subject known to have evidence of acute or chronic hepatitis B, hepatitis C or human immunodeficiency virus (HIV), or psychiatric illness/social situations that would limit compliance with study requirements or compromise the ability of the subject to give written informed consent • Clinical diagnosis of active tuberculosis • Receipt of live attenuated vaccination within 30 days prior to study entry or within 30 days of receiving durvalumab • Male patients of reproductive potential who are not employing an effective method of birth control (failure rate of less than 1% per year) • Any condition that, in the opinion of the investigator, would interfere with evaluation of study treatment or interpretation of patient safety or study results • Participation in another clinical study with an investigational product during the last 30 days before inclusion • Any previous treatment with a PD-1 or PD-L1 inhibitor, including durvalumab • Current or prior use of immunosuppressive medication within 28 days before the first dose of durvalumab, with the exceptions of intranasal and inhaled corticosteroids or systemic corticosteroids at physiological doses, which are not to exceed 10 mg/day of prednisone, or an equivalent corticosteroid • Receipt of the last dose of anti-cancer therapy (chemotherapy, immunotherapy, endocrine therapy, targeted therapy, biologic therapy, tumor embolization, monoclonal antibodies, other investigational agent) ≤ 21 days prior to the first dose of study drug or ≤ 4 half-lifes of the agent administered, whichever comes first • Previous enrollment or randomization in the present study • Involvement in the planning and/or conduct of the study (applies to both AstraZeneca staff and/or staff of sponsor and study site) • Patient who might be dependent on the sponsor, site, or the investigator • Patient who has been incarcerated or involuntarily institutionalized by court order or by the authorities § 40 Abs. 1 S. 3 Nr. 4 AMG • Patients who are unable to consent because they do not understand the nature, significance, and implications of the clinical trial and therefore cannot form a rational intention in the light of the facts (§ 40 Abs. 1 S. 3 Nr. 3a AMG)	

### Procedures for stratification

Patients are stratified by the principal investigator or authorized delegate from the study staff according to modified Cancer and Age Research Group (CARG) to receive, respectively [10, 22]:

- Total risk score ≤ 3 → doublet chemotherapy
- Total risk score > 3 → single-agent chemotherapy

The aim is to prevent > 50% of standard chemotherapy toxicities (CTCAE grade III/IV). The risk score will be determined according to Table 2.

**Table 2:** Modified CARG Risk score determination for treatment stratification (modified from Hurria et al. JCO 2011)

Toxicity factor/question	Value/response	Score
Age ≥ 72 years	≥ 72	2
Hemoglobin	< 11 g / dL (male) < 10 g/dL (female)	3
Creatinine clearance	< 34 mL/min	3
Hearing	Fair/worse	2
Number of falls in the past 6 months	1 or more	3
Taking medications	With some help or unable	1
Walking 1 block (100 m)	Somewhat limited or limited a lot	2
Decreased social activity because of physical/emotional health	Limited at least sometimes	1

### Study procedures

The subjects must first read, understand, and sign the approved informed consent form (ICF) before any study-specific screening procedures are performed. After signing the ICF, completing all screening procedures, and being deemed eligible for entry, subjects will be enrolled in the study. Procedures that are performed prior to the signing of the ICF and are considered standard of care may be used as screening assessments if they fall within the 28-day screening window (in particular tumor stagings).

After the stratification procedure (modified CARG score) done by the investigator, the investigator will access the randomization website to assign the participant to the treatment arms. Randomization is performed using permuted block randomization with fixed block lengths. Treatment arm allocation (ratio 1:1) will be done following the Standard Operational Procedures of the Institut für Klinische Forschung (IKF, Frankfurt Germany), which is the clinical research organization of the DURATION trial (CRO). After randomization the system will immediately confirm a patient’s allocation to the treatment arms, to receive either four cycles of single agent or doublet chemotherapy or two cycles of single-agent or doublet chemotherapy followed by two cycles of immunotherapy. After four cycles of standard chemotherapy, patients receive either follow-up care (arm A and D) or a maintenance therapy with durvalumab for a maximum of 2 years in the experimental arms B and C. Dose modification and toxicity management are described in detail in the “Treatment plan” section of the protocol. Furthermore, detailed information about permitted or prohibited concomitant treatment can be obtained from the protocol.

#### Subject adherence to protocol interventions

No particular methods to improve adherence to trial intervention have been implemented. Due to the nature of the disease under study (advanced or metastatic lung cancer), patients are naturally motivated to adhere to the trial intervention. Furthermore, the trial medication is not self-administered by the study subjects but rather investigator administered according to a pre-specified visiting calendar of the trial protocol.

#### Strategies to achieve target sample size

The DURATION trial was set-up with the clinical trial network of the Arbeitsgemeinschaft Internistische Onkologie (AIO). Each of the 30 participating sites was selected by the sponsor based on former and anticipated accrual performance. The accrual rate is monitored on a monthly basis and any shortfall communicated to the CI and the sponsor. Regular newsletters and meetings within the trial network are routine tools to maintain a steady accrual rate. Based on the discretion of the sponsor, additional study sites may be included to bolster recruitment.

For each patient enrolled, an electronic case report form (eCRF) must be completed by the principal investigator or authorized delegate from the study staff. This also applies to records for those patients who fail to complete the study. If a patient withdraws from the study, the reason must be noted in the eCRF. Subjects who are permanently discontinued from the study medication will be followed for safety unless consent is withdrawn or the subject is lost to follow-up or enrolled in another clinical study. All subjects will be followed for survival. Subjects who decline to return to the site for evaluations will be offered follow-up by phone as an alternative.

Treatment emergent adverse events (AEs) according to Common Terminology Criteria for Adverse Events (CTCAE) version 4.03 will be recorded in the eCRF using a recognized medical term or diagnosis that accurately reflects the event. AEs will be assessed by the investigator for severity, relationship to the investigational product, possible etiologies, and whether the event meets criteria of a serious adverse event (SAE) and therefore requires immediate notification to the CRO. AEs and SAEs will be recorded during the entire study duration, including the regular 30-day safety follow-up period after the end-of-treatment (EOT) visit. Subsequently, subjects will be followed for ongoing study treatment-related AEs until resolved, return to baseline, or deemed irreversible, until lost to follow-up, or withdrawal of study consent. Non-SAEs are recorded from time of signed informed consent until 30 days after the last dose of the investigational medicinal product (IMP). SAEs are recorded from time of signed informed consent until 90 days after the last dose of IMP. AEs of special interest (non-serious and serious AESI) are recorded from time of signed informed consent until 90 days after last dose of IMP. The investigator is responsible for ensuring that all AEs observed by the investigator or reported by patients are properly captured in the patients’ medical records. During the course of the study all AEs and SAEs should be proactively followed up for each subject. Every effort should be made to obtain a resolution for all events, even if the events continue after discontinuation/study completion.

A data safety monitoring board (DSMB) will monitor trial conduct. The primary objective of the DSMB is to monitor the safety of the intervention of the clinical study according to the protocol. The DSMB will evaluate the safety of the study intervention and will propose changes, termination, or continuation of the trial to the sponsor and the coordinating investigator. It will consist of two experienced thoracic oncologists. The first interim safety assessment will be conducted after the first 20 subjects have been treated with at least two cycles of durvalumab. Thereafter, yearly assessments, synchronized with the annual safety reports, will be performed. Details are provided in the protocol referring to the DSMB Charter.

#### Monitoring/audits

The CRO must provide a trained monitor to assist the investigators in conducting the clinical study. The monitor has the responsibility of reviewing the ongoing study with the investigators to verify adherence to the protocol and to deal with any problems that arise. The study monitor will review the eCRF data for completeness and accuracy during the monitoring visits. The study monitor will point out any discrepancies between source data and the data captured in the eCRF. The monitor will issue electronic queries to site staff to initiate discrepancy resolution. Discrepancies which require eCRF data corrections have to be re-solved by authorized site personnel by answering these monitoring queries. The frequency of on-site visits will depend on the number of recruited patients and results of prior monitoring (risk-adapted monitoring). The monitor must be given access to subjects’ medical records and other study-related records needed to verify the entries in the eCRF. The investigator agrees to cooperate with the monitor to ensure that any problems detected in the course of these monitoring visits, including delays in completing case report forms, are resolved. The investigator has to ensure that all data required according to this protocol will be entered promptly in the eCRF.

#### Collection of safety data/harms

It is the responsibility of the investigators to document all AEs occurring during the study in the patients’ medical files and eCRFs. Any SAE or AE of special interest, overdose of IMP, and pregnancies must be reported immediately (within 24 h) to the sponsor. The sponsor and the CRO will ensure compliance with all regulatory reporting requirements, including the notification of the appropriate Ethics Committees, Competent Authority, and participating investigators of all SAEs occurring at the sites in accordance with national law, ICH Good Clinical Practice, and European/EMA requirements.

A sponsor representative (e.g., CRO) will medically review all SAE reports and perform the expectedness assessment.

Every SAE assessed by either the investigator or the sponsor as suspected to be related to IMP and assessed as being either unexpected or unexpected with regard to outcome or severity of the event will be reported by the sponsor as suspected unexpected serious adverse reaction (SUSAR) to the competent authority, responsible ethics committee, and investigators of the trial in line with the national regulations in effect (German drug law [AMG] and GCP-V § 13).

### Data management and data quality assurance

Accurate and reliable data collection will be assured by verification and cross–check of the eCRF against the investigator’s records by the study monitor (source document verification), and the maintenance of a drug-dispensing log by the investigator. Data for this study will be recorded via eCRF by the site from the source documents. Data are reviewed and checked for omissions, apparent errors, and values requiring further clarifications using computerized (automatic) and/or manual procedures. Data queries requiring clarification are communicated to the site for resolution via the CRO. Only authorized personnel will make corrections to the clinical database and an audit trail will document all corrections.

#### Information flow

Protocol amendments are submitted to the competent authorities and ethics committees for approval according to local legislation. Only after regulatory approval changes to the protocol are communicated to all sites via mail or newsletter. Important safety information (e.g., SUSARS) are communicated directly to the participating investigator in parallel to reporting such information to the competent authorities.

An overview of all study procedures is presented in Table 3.

**Table 3:** Schedule of assessments

						Arms B and C	All arms	
Procedure/assessment	Screening 28d before C1D1	Treatment (q3w) ±2d				Maintenance (q4w) ±7d	End of treatment	Follow-Up ±7d
	Inclusion	C1 – C2 (Arm B/C) C1 - C4 (Arm A/D)		C3 (Arm B/C)	C4 (Arm B/C)	C5 – Cx		
		Day 1	Day 8					
Informed consent, eligibility criteria, demographics, medical and disease history	x							
FFPE tumor tissue (PD-L1)	x							
Vital signs, physical examination	x	x	x	x	x	x	x	
ECOG	x			x		x	x	x^a^
AE/SAE	x	x	x	x	x	x	x	x
CT/MRI of tumor lesions	x			x		x^b^		x^c^
HR-QoL	x			x		x^b^	x	x
Charlson Comorbidity Index	x							
CARG-score	x							
Geriatric assessments	x					x	x	x^d^
Biomarker sample		x^e^		x	x^f^	x^g^	x	x^a^
**Treatments**								
**Arm A**		CHT	CHT	CHT	CHT			
**Arm B**		CHT	CHT	Durvalumab	Durvalumab	Durvalumab		
**Arm C**		CHT	CHT	Durvalumab	Durvalumab	Durvalumab		
**Arm D**		CHT	CHT	CHT	CHT			

^a^ At time of PD

^b^ Every other cycle (every 8 weeks)

^c^ During follow-up, CTs or MRIs will be performed every 8 weeks (±7 days) until confirmed disease progression or death in the context of standard care

^d^ At FU1 and time of PD

^e^ At baseline (C1D1)

^f^ Only arm B and C

^g^ After two cycles for durvalumab maintenance

##### Treatment arms A and D: standard of care single agent or doublet chemotherapy

Arm A: *nab*-paclitaxel 100 mg/m^2^ on days d1 and d8 and carboplatin AUC 5 on day 1, every 3 weeks up to four cycles.

Arm D: gemcitabine 1000 mg/m^2^ on days d1 and d8, given every 3 weeks, or vinorelbine 30 mg/m^2^ on days d1 and d8 every 3 weeks up to four cycles.

##### Treatment arms B and C: two cycles of single agent or doublet chemotherapy followed by durvalumab

Arm B: two cycles of *nab*-paclitaxel 100 mg/m^2^ on days d1 and d8 and carboplatin AUC 5 on day 1, every 3 weeks followed by durvalumab 1125 mg every 3 weeks for two cycles followed by maintenance with durvalumab 1500 mg every 4 weeks.

Arm C: two cycles of gemcitabine 1000 mg/m^2^ on days d1 and d8, given every 3 weeks or vinorelbine 30 mg/m^2^ on days d1 and d8 every 3 weeks up to four cycles followed by durvalumab 1125 mg every 3 weeks for two cycles followed by maintenance with durvalumab 1500 mg every 4 weeks.

##### Tissue and blood collection for exploratory endpoints

Tissue collection

For each patient a FFPE tumor tissue block (archival or recent) or a minimum of ten unstained slides of tumor sample (2–3-μm sections; slices must be recent and collected on slides provided by the sponsor) must be available for biomarker (PD-L1) evaluation as stated in the inclusion criteria. Biopsy should be excisional, incisional, or core-needle. Fine-needle aspiration is insufficient. Tumor PD-L1 expression is measured by an immunohistochemistry assay using SP263 antibody. If a re-biopsy upon tumor progression under study treatment is performed, submission of this tumor material is highly valued.

Blood collection

Participation of patients in the biomarker program is voluntary and must be documented in the informed consent form. The time points for blood sampling are before start of any treatment at baseline and after two cycles of chemotherapy, as well as after 20 weeks of study participation for all patients with stable disease or tumor regression and at the time point of detection of tumor growth in patients with disease progression. In arms B and C, blood is additionally collected after the first cycle of durvalumab.

#### Study endpoints

##### Primary endpoint

The primary endpoint is the rate of treatment-related grade III/IV adverse events (CTCAE V4.03). It will be calculated taking into account patients who have received at least one dose of study medication.

##### Secondary endpoints

Secondary endpoints will be:

- Overall response rate (ORR) according to RECIST 1.1 criteria
- Progression-free survival (PFS), calculated from the date of subject randomization until the date of confirmed PD or death from any cause; if no event is observed (e.g., lost to follow-up) PFS is censored at the time of last tumor assessment
- Overall survival (OS) will be calculated from the date of subject randomization until the date of death from any cause; if no event is observed (e.g., lost to follow-up), OS is censored at the day of last subject contact
- AEs/SAEs according to CTCAE 4.03
- Health-related quality of life (HR-QoL) assessment using Functional Assessment of Cancer Therapy–Lung (FACT-L) questionnaire, a standard instrument to determine QoL including lung-specific domains [23]

##### Exploratory endpoints

Exploratory analysis on tissue samples

Patients will provide a tumor tissue sample at screening to determine PD-L1 expression level. This assessment will be centralized and performed by an immunohistochemistry assay using SP263 antibody. The results will be used to correlate PD-L1 staining intensity (proportion of positive tumor and immune cells) with durvalumab efficacy.

Exploratory analysis on blood samples

Blood samples that are collected at different time points will be used to characterize the immune response and investigate biological processes before, during, and after the administration of the treatment. Flow cytometric (FCM) analysis will be used to characterize the immune response and the biological processes before, during, and after the administration of the treatment. Whole blood samples will be analyzed with this modality with respect to changes in T-cell composition. Abundances of immunostimulatory cytokines will be quantified by measuring serum pro- and anti-inflammatory cytokines. Analysis of mutational load on cfDNA will be performed.

### Statistical analysis

The primary safety endpoint for the study is the occurrence of CTCAE grade III/IV toxicities assessed from the first dose to 90 days after the last dose of durvalumab. This is also the primary study endpoint on which the sample size calculation is based. According to the results presented at ASCO 2015 by Rizvi et al., it is assumed that the probability for a CTC grade III/IV toxicity for patients from the pooled experimental arms B + C receiving durvalumab amounts to P_B + C_ = 0.18 [24]. Based on reported data of selected treatment-related AEs (combination chemotherapy *nab*-paclitaxel/carboplatin [25], mono-chemotherapy gemcitabine/vinorelbine [7]), it is furthermore assumed that the rate of patients with a CTC grade III/IV toxicity in the pooled control arms A + D receiving chemotherapy only amounts to P_A + D_ = 0.35. With the planned number of patients of *N* = 200, the assumed difference between these two groups can be detected using a Chi-square test at a two-sided significance level of α = 10% with a probability of 1 − β = 0.80, also taking into account a dropout rate of 15%. Sample size calculation was performed using ADDPLAN v6.1.

It should be noted that the study is not powered to detect significant differences with regard to the efficacy endpoints, since its primary aim is to assess safety and tolerability. Hence, no confirmatory evidence can be drawn from the efficacy evaluation. Accordingly, all *p* values for efficacy outcomes are only to be interpreted descriptively and no adjustment for multiple testing will be done.

The null hypothesis for the primary (safety) endpoint of the trial is defined as H_0_: P_B + C_ = P_A + D_ (i.e., the rate of patients with a CTC grade III/IV toxicity is equal in the pooled experimental arms B + C and the pooled control arms A + D), which is tested against its alternative H_1_: P_B + C_ ≠ P_A + D_ (i.e., there is a difference between the pooled experimental arms B + C and the pooled control arms A + D with regard to the rate of patients with a CTC grade III/IV toxicity). These hypotheses will be assessed at a two-sided significance level of α = 0.1 using a Mantel-Haenszel Chi-square test adjusting for the stratum “adopted combination/not prone to combination”. Missing data for the primary outcome variable will be replaced by using multiple imputation [26]. The analysis of the primary endpoint will be based on the safety population comprising all patients enrolled who received at least one dose of study medication. Secondary endpoints will be analyzed descriptively. The analysis of PFS will be performed analogously to the analysis of OS by calculating 1-year and 2-year rates and median times per group, conducting a stratified log rank test, calculating Kaplan Meier curves, and estimating the hazard ratio using a Cox regression adjusting for the stratum “adopted combination/not prone to combination”. Other secondary endpoints will be analyzed descriptively by tabulating the measures of the empirical distributions. Subgroup analyses according to PD-L1 expression will be performed. A detailed methodology for the statistical analysis will be described in the statistical analysis plan (SAP), which will be finalized before data base lock. Statistical analysis will be done using SAS v9.4 or higher (SAS Institute, Cary, NC, USA).

## Discussion

Lung cancer is the most common cause of cancer-related death worldwide and it is predominantly a disease of the elderly, with about 50% of patients diagnosed aged 70 years or older and with about 14% of these being older than 80 years [2]. Due to the fact that lung cancer is mostly diagnosed at an advanced stage, prognosis is very poor.

Chemotherapy is effective in elderly NSCLC patients. However, they might experience treatment toxicity and deterioration due to side effects. The Elderly Selection on Geriatric Index Assessment (ESOGIA) trial was the first prospective study to investigate comprehensive geriatric assessment (CGA) incorporation into cancer treatment decisions and its impact on survival outcomes [27]. The study randomly assigned 192 stage IV NSCLC patients with a median age of 77 years to a standard arm or a CGA arm, where patients received either one of two chemotherapy regimens or best supportive care (BSC) based on performance status (PS) and age or on the CGA evaluation, respectively. Importantly, the treatment allocation based on CGA reduced treatment toxicities and the number of toxicity-related treatment failures, although it was not able to improve treatment failure-free survival or OS. This trial for the first time demonstrated the feasibility of incorporating CGA in a multicenter clinical trial setting and that CGA-based treatment is associated with decreased toxicity in elderly NSCLC patients. In clinical practice, however, the implementation of CGA has been difficult because it is rather time- and resource-consuming. Consequently, alternative pre-therapy risk assessment tools have been developed to predict chemotherapy toxicity, the CRASH and CARG scores being the two most promising tools for assigning patients to varying chemotherapy intensities based on pre-therapy risk assessment.

In the DURATION trial, the CARG toxicity prediction tool will be used to guide treatment intensity with the intention to improve outcomes of elderly and frail patients. The CARG score has been developed to stratify patients and identify those at higher risk for chemotherapy toxicity [10]. It consists of 11 questions, including five geriatric assessment questions and six clinical questions concerning items retrieved from everyday practice. The CARG score was validated in lung cancer, showing its value in better distinguishing the risks of chemotherapy toxicity in older patients compared to the Karnofsky performance status (KPS) [28]. Its value in treating and predicting mortality in elderly patients with cancer is now broadly accepted. Minor modifications of the CARG score in the DURATION trial include the removal of the default scoring items “polychemotherapy” and standard dose as well as the items “GI” or “GU cancer”, which do not apply to this study. The predictive properties of the CARG score remain unchanged.

According to this modified CARG toxicity tool, patients in the DURATION trial will be classified as “fit” or as “less fit” with regard to receiving a platinum-based combination chemotherapy. “Less fit” patients will be treated with a single-agent chemotherapy of either vinorelbine or gemcitabine. Both single-agent chemotherapy regimens were established as the standard of care over best supportive care for first-line therapy of advanced NSCLC patients aged 70 years or older [5, 29]. Patients that are stratified as fit will receive treatment according to current ESMO guidelines for advanced NSCLC that recommend platinum-based combination chemotherapy for patients aged > 70 years with PS 0–2 and adequate organ function based on a recent systematic review [30]. The combination chemotherapy applied in the DURATION trial consists of a combination of *nab*-paclitaxel with carboplatin as inferred from clinical trials and retrospective analyses that demonstrated superiority of carboplatin/*nab*-paclitaxel over carboplatin/paclitaxel with respect to efficacy and safety in elderly patients [25, 31]. Both patient groups treated with either single-agent or doublet chemotherapy will be subjected to randomization for treatment with the PD-L1 inhibitor durvalumab.

Based on promising results from clinical trials, immuno-oncology agents such as PD-1 - or PD-L1 inhibitors have found their way into frequent clinical use, even in the first-line setting, and have revolutionized the treatment landscape of NSCLC [32]. However, due to underrepresentation of older patients in large trials that led to approval of checkpoint inhibitors, all available efficacy and safety data for this patient group is derived from subgroup analyses. Such analyses of second-line trials revealed no differences in response rates and survival between patients aged less or more than 65 years [12, 14, 18, 33, 34] . Similarly, KEYNOTE-024, a first-line clinical trial comparing pembrolizumab with combination chemotherapy in advanced NSCLC patients with PD-L1 expression > 50%, indicated no differences in the beneficial effect of pembrolizumab when comparing patients aged < 65 years and > 65 years) [16]. Of note, no differences regarding toxicities between age groups were observed [14]. However, to date no data from randomized phase III trials assessing the efficacy of PD-1/PD-L1 targeting agents in elderly patients with advanced NSCLC are available.

In addition, addressing immuno-oncology agents specifically in older patients is of particular interest as a phenomenon called immunosenescence has to be considered. This age-related decline in the immune system includes reductions in B- and T-cell proliferation and function, quantitative differences in cellular subsets, functional impairment, and qualitative changes in APCs and an accumulation of regulatory T cells—processes that eventually could be associated with impaired immune response to pathogens and tumor cells [18].

Considering the growing number of immune checkpoint inhibitors that are available for the treatment of NSCLC patients, it is also important to learn about potential differences between PD-1- and PD-L1- targeting agents. Although each drug has shown activity in NSCLC, comparing these agents in terms of efficacy and toxicity is the subject of current research. A recent systematic review of clinical trials that tested both PD-1 and PD-L1 antibodies did not find significant differences between the two types of checkpoint inhibitors regarding the reported response rates and toxicity profiles [35]. The most notable difference was observed regarding grade III/IV immune-mediated pneumonitis that was slightly higher with PD-1 inhibitors compared with PD-L1 inhibitors. This could possibly be explained by the fact that anti-PD-L1 antibodies still allow for the interaction of PD-1 with its other ligand, PD-L2, thus resulting in a weaker blockade of the negative inhibitory signal and reduced autoimmunity [35]. The development of autoimmune pneumonitis has to be carefully monitored in NSCLC patients as this has led to a few treatment-related deaths in early-phase studies of PD-1-targeting agents [13, 36, 37] and patients with lung cancer are more vulnerable to toxicities given the older age of the patient population and the presence of comorbid conditions. Given the putatively lower risk of developing autoimmune-mediated toxicities with PD-L1 targeting agents, the use of durvalumab, a selective, high-affinity, human IgG1 monoclonal anti-PD-L1 antibody [38–40], is expected to be more suitable for treatment of a more vulnerable patient group such as old or frail NSCLC patients that are included in the DURATION trial. Encouraging antitumor activity of durvalumab has already been shown in an early-phase clinical study involving multiple advanced solid tumors, including NSCLC, and recently it has been approved for patients with locally advanced NSCLC after chemoradiotherapy [38, 41].

In the DURATION trial, checkpoint inhibition by durvalumab is combined sequentially with cytotoxic chemotherapy. It is thought that modulation of the immune response through PD-1 inhibition may be enhanced by the potential immunogenic effects of cytotoxic chemotherapy, e.g., by increasing the potential for antigen cross-presentation by dendritic cells after the destruction of tumor cells, inhibiting myeloid-derived suppressor cells, increasing the ratio of cytotoxic lymphocytes to regulatory T cells, and blocking the STAT6 pathway to enhance dendritic-cell activity [42–45]. Thus, the two cycles of induction chemotherapy that are applied in the DURATION trial are expected to lead to a prompt disease-stabilizing effect, which can be efficaciously extended by a consecutive PD-L1 immunotherapy with durvalumab.

Current treatment guidelines recommend the use of immunotherapy alone or in combination with CT also for older lung cancer patients with adequate PS ECOG [46]. Considering the underrepresentation of older and frail patients in main pivotal trials that led to the approval of these new treatment modalities, there’s a lack of data about safety and efficacy in this group. Aging processes, comorbidities, and undetected frailty could affect treatment tolerance in the face of a poor clinical benefit. The DURATION trial will help to close the current gap in knowledge about safety and tolerability of checkpoint inhibitors in elderly and frail lung cancer patients, an important cohort that has been underrepresented in clinical trials for too long.

## Trial status

Patient accrual started in December 2017 with protocol version 5.0 and is currently ongoing according to the protocol version number 7. Two amendments were necessary, due to annual updated IB of Durvalumab, leading to protocol version 6.0 (approval 24.05.2018) and version 7.0 (approval 27.06.2019). At present, 30 centers are participating in this study. The approximate end of recruitment will be in December 2020.

## Supplementary information

**Additional file 1.** SPIRIT 2013 Checklist: Recommended items to address in a clinical trial protocol and related documents.

## Data Availability

Not applicable as no primary data are contained, generated, or analyzed.

## Abbreviations

AE: Adverse event
AIO: Arbeitsgemeinschaft Internistische Onkologie
APC: Antigen presenting cells
ASCO: American Society of Clinical Oncology
AUC: Area under the curve
BSC: Best supportive care
CARG: Cancer and aging research group
CCI: Charlson Comorbidity Index
CD: Cluster of differentiation
cfDNA: Cell-free deoxyribose nucleic acid
CGA: Comprehensive geriatric assessment
CRASH: Chemotherapy Risk Assessment Scale for High-Age Patients
CT: Computed tomography
CTCAE: Common Terminology Criteria for Adverse Events
ECOG: Eastern Cooperative Oncology Group
EGFR: Epidermal growth factor receptor
EML4ALK: Echinoderm microtubule-associated protein-like 4 anaplastic lymphoma kinase
ESMO: European Society for Medical Oncology
FACS: Fluorescence activated cell sorter
FFPE: Formalin-fixed, paraffin-embedded
GI: Gastrointestinal
GU: Genitourinary
G8: Geriatric 8
HBV: Hepatitis B virus
HCV: Hepatitis C virus
HR-QoL: Health related quality of life
IHC: Immunohistochemistry
IMP: Investigational medical product
irRECIST: Immune-related response evaluation criteria in solid tumors
MRI: Magnetic resonance imaging
6MWT: Six-minute walk test
NSCLC: Non-small cell lung cancer
ORR: Objective response rate
OS: Overall survival
PD-1: Programmed death-1
PD-L1: Programmed death-ligand 1
PFS: Progression-free survival
RECIST: Response evaluation criteria in solid tumors
SAE: Severe adverse event
QoL: Quality of life
